# Genome editing via non-viral delivery platforms: current progress in personalized cancer therapy

**DOI:** 10.1186/s12943-022-01550-8

**Published:** 2022-03-11

**Authors:** Tianxia Lan, Haiying Que, Min Luo, Xia Zhao, Xiawei Wei

**Affiliations:** 1 Laboratory of Aging Research and Cancer Drug Target, State Key Laboratory of Biotherapy, National Clinical Research Center for Geriatrics, West China Hospital, Sichuan University, No. 17, Block 3, Southern Renmin Road, Sichuan 610041 Chengdu, China; 2 Department of Gynecology and Obstetrics, Development and Related Diseases of Women and Children Key Laboratory of Sichuan Province, Key Laboratory of Birth Defects and Related Diseases of Women and Children, Ministry of Education, West China Second Hospital, Sichuan University, Sichuan 610041 Chengdu, China

**Keywords:** Genome editing tool, Personalized anti-cancer therapy, Non-viral delivery system

## Abstract

Cancer is a severe disease that substantially jeopardizes global health. Although considerable efforts have been made to discover effective anti-cancer therapeutics, the cancer incidence and mortality are still growing. The personalized anti-cancer therapies present themselves as a promising solution for the dilemma because they could precisely destroy or fix the cancer targets based on the comprehensive genomic analyses. In addition, genome editing is an ideal way to implement personalized anti-cancer therapy because it allows the direct modification of pro-tumor genes as well as the generation of personalized anti-tumor immune cells. Furthermore, non-viral delivery system could effectively transport genome editing tools (GETs) into the cell nucleus with an appreciable safety profile. In this manuscript, the important attributes and recent progress of GETs will be discussed. Besides, the laboratory and clinical investigations that seek for the possibility of combining non-viral delivery systems with GETs for the treatment of cancer will be assessed in the scope of personalized therapy.

## Background

Cancer is a very prevalent disease and is also the leading cause of death worldwide [1]. According to *Global Cancer Statistics 2020*, there were about 19.3 million new cancer cases in 2020. Moreover, an estimated 10.0 million cancer deaths occurred in the same year [2]. Besides, the incidence and mortality of cancer are growing year by year [3–5]. Therefore, to control the growing mortality and incidence of cancer more effectively, innovative and potent therapeutic methods need to be developed [6]. Up to date, a series of novel anti-cancer approaches have been proposed [7]. They are characterized by improved efficiency and safety, such as personalized therapy [8], immunotherapy [9], targeted therapy [10], combination therapy [11], and gene therapy [12]. Among them, personalized therapy is distinct because its development does not heavily rely on the major technological breakthrough in a specific field but requires the harmonious collaboration of multiple disciplines including diagnostics, genome sequencing, target screening, and treatment designing [13]. Since the carcinogenic factors (e.g., oncogenes, cancer stem cells) are different from patients to patients [14, 15], personalized therapies present themselves as a promising solution for tumor heterogeneity because they make it possible to precisely destroy or fix the neoplastic genes based on the comprehensive genomic analyses of different patients and tumors [16].

Genome editing is an ideal way to implement personalized anti-cancer therapy because it provides the possibility of directly modifying the pro-tumor genes [17]. In addition, genome editing can also be used to develop personalized immunotherapies by reprogramming the immune cells [18]. In comparison to early gene engineering methods that randomly insert genes into the host genome [19], genome editing is carried out by precisely inserting, deleting, modifying or replacing DNA or RNA sequences at specific sites in the genome [20, 21]. Generally, the procedure of most genome editing mechanisms consists of three steps: recognition, cleavage, and repair [22]. The genome editing tools (GETs) could precisely recognize the target site where the double strand break (DSB) would then be generated [23]. Subsequently, the DSB would be repaired by homology-directed repair (HDR) or nonhomologous end-joining (NHEJ) [24]. In particular, HDR mediates the insertion or replacement of genes [25], while NHEJ induces the disruption of genes [26]. The four major genome editing platforms include mega-nucleases, zinc-finger nucleases (ZFNs), transcription activator-like effector nucleases (TALENs), and clustered regularly interspaced short palindromic repeats (CRISPR)/Cas9 [27, 28]. While all of them have been considered as potential weapons in the battle against cancer, the accurate [29], convenient and effective CRISPR-Cas9 system is the most advanced one [30]. Additionally, the prerequisite for successful genome editing is the efficient delivery of GETs into the cell nucleus [31].

The delivery systems for genome editing therapies are commonly classified into viral delivery systems and non-viral delivery systems [32]. Although viral vectors are widely used for the delivery of gene therapies [33–35], they are associated with flaws like carcinogenesis [36], genotoxicity, immunogenicity [37], and insertional mutagenesis [38]. Compared to viral delivery systems, non-viral delivery systems have fewer safety concerns [39]. Furthermore, non-viral approaches can carry larger GETs and provide more stable control over the duration time the GETs stay in cells [40]. Therefore, it is conceivable that combining GETs with non-viral delivery systems may be a prospective strategy for the personalized and targeted cancer medicine. In this review, the important attributes and recent progress of GETs will be evaluated in terms of mechanisms and genome editing efficiency. In addition, the studies that combined non-viral delivery systems with GETs for the treatment of cancer will be assessed. Further, the clinical trials designed to appraise the efficacy and safety of anti-cancer therapies which combine GETs with non-viral delivery systems will be discussed according to published information.

### Genome editing in the development of personalized anti-cancer therapies

The 21^st^ centuray has witnessed spectacular upgrades of anti-cancer approaches, being genome editing-based personalized therapy deemed as one of the most promising strategies. Mega-nuclease, ZFN, TALEN, and CRISPR-Cas9 are the currently major genome editing platforms. While mega-nucleases are considered as the earliest GETs, ZFNs and TALENs manifest higher feasibility and editing efficiency. However, boosted by the unparalleled simplicity and convenience, CRISPR-Cas9 system has become the most prevelantly used GET at present. Moreover, since genome editing can be leveraged for the disruption or correction of oncogenes [41, 42], reprogramming of anti-cancer immune cells [43], and establishment of cancer models [44], it emerge as a powerful and versatile tool in the development of anti-cancer therapies.

#### Mega-nucleases

**Fig. 1:**
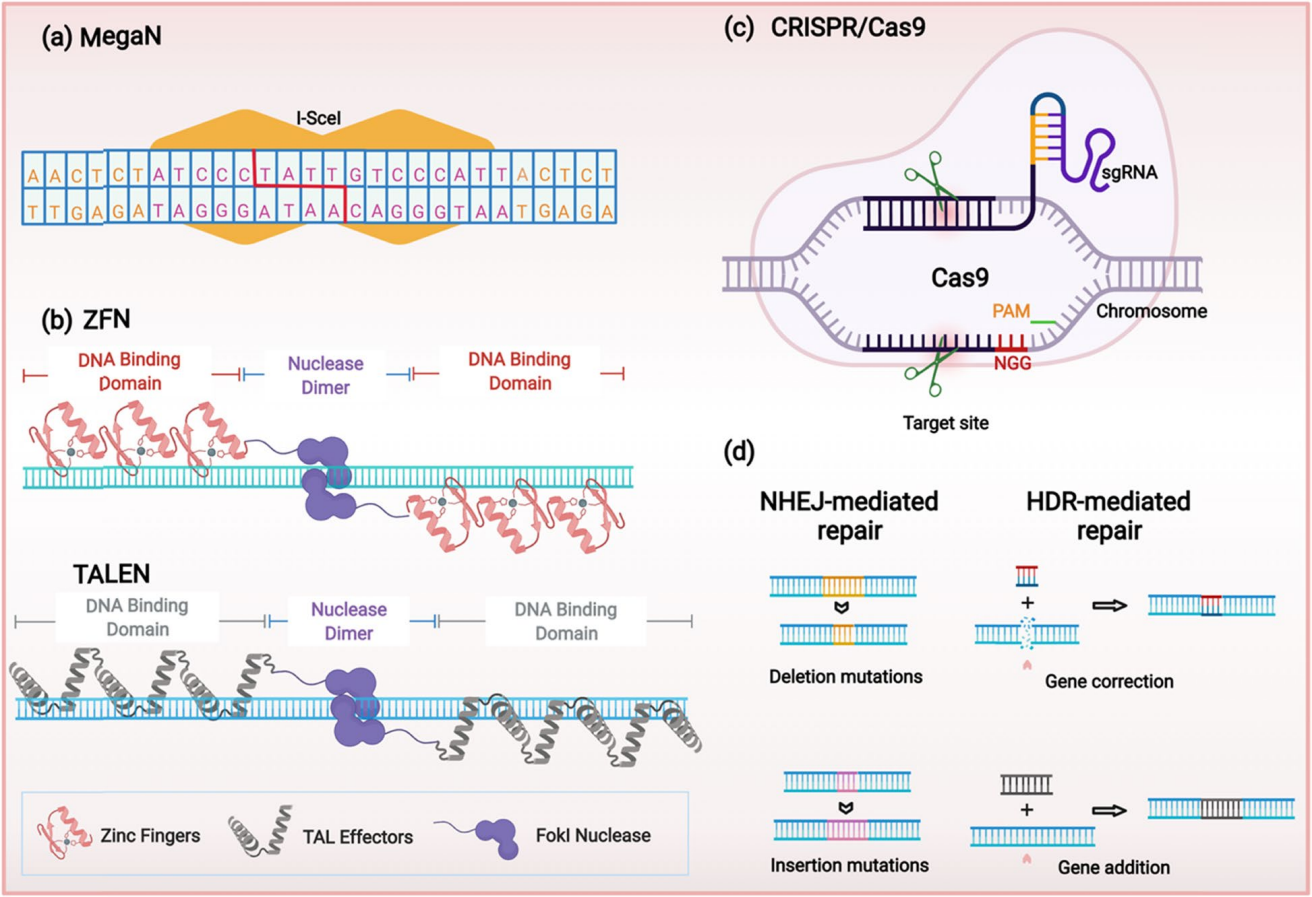
Schematic diagram of the four GETs and the basic mechanisms of genome editing. **a** I-SceI: one of the most popular mega-nucleases. **b** The DNA binding domain of ZFNs and TALENs are both modular, the Fokl nuclease can create DSB. **c** CRSPR/Cas9 system comprises a Cas9 endonuclease and a sgRNA that target the sequence next to PAM site. **d** Following the creation of DSB, it would be repaired by HDR or NHEJ

Mega-nuclease is a family of endodeoxyribonucleases that are characterized by a large (12-40 bps) recognition site [45]. Based on sequential and structural characteristics, the family could be divided into five sub-groups: LAGLIDADG, GIY-YIG, HNH, His-Cys box and PD-(D/E)XK [46]. Among them, the nucleases in the LAGLIDADG family were the most used and well-studied GETs [47]. Notably, mega-nucleases were also the first biological molecules being used for precise modification of genes, which, in another word, led to the arrival of genome editing era [48]. LAGLIDADG proteins have two important properties: first, they could engage in the splicing of their own introns as RNA maturases; second, they could recognize and cut the exon-exon junction sequence wherein their introns could be inserted [49]. In addition, two of the most popular LAGLIDADG proteins are I-SceI (Fig. 1a) and I-CreI [50]. Their abilities to recognize and cleave the target genes allow them to exert therapeutic functions such as gene correction and insertion of therapeutic genes.

However, the further applications of mega-nucleases in personalized anti-cancer therapies are being hampered by several drawbacks. Firstly, given the fact that all types of cancers are caused by certain oncological mutations, such genetic variations differ from patients to patients [51–53]. But natural mega-nucleases could only create DSBs in the specific recognition sites that do not exist in oncological genes [45], which means it is difficult to apply such natural mega-nucleases for the correction of cancer-associated mutations. Nevertheless, this problem could be overcome by the engineered mega-nucleases, as they can target sequences other than natural recognition sites with considerable efficiency. For example, it has been reported that, by using I-CreI of LAGLIDADG family as scaffold, the engineered mega-nuclease could target human RAG1 gene and induce the homologous recombination in 6% of transfected human cells [54]. Hence, the development of mega-nuclease engineering techniques would provide more feasibilities in terms of personalized anti-cancer therapies. Secondly, the gene targeting efficiency is relatively low. Reportedly, the highest targeting efficiency of mega-nuclease is 66% in human 293H cell line [55], however, a majority of studies just obtained 1%-20% efficiency in human cells [56–59]. Besides, the off-target issues are also hampering the further translation of mega-nuclease-based applications [60, 61], especially for the personalized anti-cancer therapies like correction of personal somatic mutations that cause cancers [62].

#### Zinc-finger nucleases

ZFN was first discovered in 1985 from Xenopus oocytes [63]. The consecutive structural analysis revealed that it comprised a site-specific DNA binding domain and a cleavage domain, and both of two are loaded on a zinc-finger (Fig. 1b). The abilities of ZFNs to recognize specific DNA sequence and create DSB make it an ideal platform for genome engineering [64]. Theoretically, the zinc finger protein can be designed to target any sites of genome [65]. In addition, such genomic modifications could be achieved through NHEJ and HDR [66]. While the NHEJ is mainly used to perform gene knockout, HDRs induced by ZFNs could be leveraged for gene correction or gene addition if proper templates were provided [64]. For example, to conduct gene knockout through NHEJ, Liu et. al. have successfully targeted and cleaved three independent genes using ZFNs. However, the frequencies of the obtained knockouts were above 1% [67]. Also, it was reported that, by inducing HDR using ZFNs and donor DNA, the “pro-stemness” gene was added to the genome of human embryonic stem cells with a frequency of 5% [68]. Hence, the abilities to elicit gene knockout, gene correction, and gene addition make ZFNs potential tool for personalized anti-cancer therapies.

ZFNs could be applied in the treatment of neoplastic diseases in three different ways. First, by specifically introducing DSBs, ZFNs could knockout oncogenes. However, Shankar et. al. have demonstrated that the ZFN-mediated editing efficiencies in some cancer cells lines were low (negligible – 6%). Besides, a substantial level of off-target events were detected by in silico analysis of DNA binding prediction [69]. These results indicate that more efforts need to be devoted to improving the targeting specificity and editing efficiency of the ZFNs used in this study. Although positive results have yet to be obtained from studies using ZFNs to knockout pro-tumor genes, this strategy remain to be promising for personalized anti-cancer therapy because the genetic backgrounds and the mutations that lead to cancers are different from patients to patients. Second, ZFNs could also be used to inhibit etiological factors of cancer like human papillomavirus (HPVs). Reportedly, the antitumor effect of the combination therapy comprising HPV E7-targeting ZFNs and chemotherapy has been evaluated. According to the report, the ability of the combination therapy to inhibit the cancer cell viability was much higher than that of chemotherapy alone. Moreover, the combination therapy also significantly suppressed the tumor growth in xenograft models [70]. Third, in anti-cancer immunotherapies, ZFNs could be used to edit the genome of immune cells to enhance their tumor-inhibitory capacities [71]. For instance, it has been shown that, knocking out the gene encoding programmed cell death-1 (PD-1) in tumor infiltrating lymphocytes (TIL) through ZFN-mediated genome editing before adoptive cell transfer leads to a 76% reduction in PD-1 surface-expression. Collectively, ZFN technology is another tool for genome editing-based anti-cancer therapies. It also holds considerable promise for the development of personalized anti-cancer therapies because it could be modified to target variable sites on the genome with favorable feasibility.

#### Transcription activator-like effector nucleases

Transcription activator-like effector nuclease (TALEN) system is established by fusing transcription activator-like effector (TALE) with DNA cleavage domain [72]. Like ZFNs, TALENs also need to be engineered to target different DNA sites. The TALE comprises three domains: a carboxyl-terminal domain, an amino-terminal domain, and a DNA-binding domain which contains a region that it is capable of conferring specificity to adenine (A), cytosine (C), guanine (G), and thymine (T) [73]. Moreover, although the DNA binding domain of ZFNs and TALENs are both modular (Fig. 1b). Each module of a TALE recognizes one nucleotide, while the recognition mediated by each zinc-finger module needs 3 nucleotides [74]. Hence, by reorganizing the TALE modules, researchers could engineer TALENs with more convenience. Besides, thanks to the previous studies on mega-nucleases and ZFNs, substantial understandings with respect to the optimization and application of such GETs have significantly facilitate the development of and technical maturation of TALEN system [72]. So far, the TALEN-mediated genome modification has been successfully performed in a variety of animals and cells [75–80].

Equipped with the superior properties such as higher efficacy, cheaper price, and improved feasibility, TALEN emerges as a better GET for personalized cancer therapeutics than ZFN [81]. However, the anti-tumor strategies of TALEN- and ZFN-based therapies are similar. For example, it was reported that the expression of IL-6 in hepatocellular carcinoma could be disrupted by TALENs. Furthermore, by analyzing the genome edited cancer cells, it was demonstrated that IL-6 promotes apoptosis and facilitate the expression level of IL-33 and VEGF-A. Finally, it was also shown that combining the genome editing with sorafenib and/or IFNα therapy significantly increased the anti-tumor effects [82]. This study suggests that TALEN could be used as personalized monotherapy or combined with other targeted therapies for the treatment of cancer. The TALEN-mediated gene disruption can also help to decipher the carcinogenic trajectories in different individuals. Moreover, TALENs were also implemented to target HPV E7. It was observed that the editing efficiency of E7 gene in cervical cancer cells was around 10-12%. In addition, the cell death induced by TALEN editing was shown to be tightly associated with cell necrosis [83]. Interestingly, in the previously mentioned study that used ZFNs to target HPV E7, the cell deaths were induced by apoptosis [70]. This discrepancy indicates that the consequences of TALEN- and ZFN-mediated knockout in cervical cancer cells might be differential, and it would be interesting to explore the mechanisms that cause this discrepancy. Furthermore, TALENs can also be used to modulate the phenotype of immune cells. Menger et. al. have performed TALEN-mediated knocking out of gene encoding PD-1 in tumor-reactive lymphocytes (TRLs) to confer resistant to PD-1 signaling on TRLs. This approach effectively enhanced the persistence of melanoma-reactive CD8 positive T cells and fibrosarcoma-reactive T cells at tumor sites [84]. In summary, the current applications of TALENs and ZFNs in cancer therapies are sharing similar mechanisms. Nevertheless, the improved module-recognizing specify, and the convenience of TALEN-mediated targeting makes them better option for developing personalized anti-tumor strategies.

#### CRISPR-Cas9 system

The first discovery of CRISPR dates back to 1987. Ishino and his colleague identified a sequence that contains five homologous sequences of 29 nucleotides, and these homologous sequences are separated by spacers [85]. Unfortunately, this study did not illustrate the biological significance of CRISPR. Later on, similar regularly spaced repeated sequences have been identified in a series of studies [86]. As a consequence, increasing attentions were focused on CRISPR, and its values in genome editing have been unveiled [87]. The natural CRISPR-Cas system acts as an adaptive immune system in prokaryotes. It could protect prokaryotes from phage infection by storing memory in host chromosomes. It consists of viral DNA and repetitive nucleotide sequences surrounding the viral DNA. These repetitive nucleotide sequences are termed direct repeats, they are surrounded at the near end by sequences encoding proteins called Cas proteins. In addition, the guide RNA of this system can be artificially manipulated for the targeting of different genes. Comparing to TALEN, CRISPR-Cas9 system is way more cost effective, it is 3-6 fold cheaper per reaction [88]. Furthermore, the generation and modification of gRNAs are also more convenient [89]. Therefore, CRISPR-Cas9 system is now deemed as the prior option for performing genome editing in laboratory studies. However, impaired by the affinity of the single guide RNA recognition, off-targeting effect is a major drawback that hinders the clinical translation [90].

The genome editing of CRISPR-Cas9 system is mediated by its two components: a Cas9 endonuclease and a single-stranded guide RNA (sgRNA) [91]. The sgRNA can recognize and bind to target sites, and the Cas9 endonuclease can then cleave the DNA (Fig. 1c). Moreover, it has been well documented that the cutting site of Cas9 endonuclease is 3 base pairs upstream of an “NGG” protospacer adjacent motif (PAM). Following the cleavage, the DSB would be repaired by NHEJ or HDR (Fig. 1d) [92].

Moreover, some recent progress made in CRISPR-Cas9-based techniques have paved the way for the development of personalized therapies. In 2016, David. Liu et. al. developed an adenine base editor system which can induce the conversion from adenine (A) to guanine (G) on DNA through rearranging the atoms, thereby easily installing point mutations in cells. In another word, this first version of base editor system can change A•T base pair into G•C base pair [93]. In 2017, the same group developed the upgraded version of base editor that can convert C:G base pair into T:A base pair with higher efficiency and product purity [94]. In the same year, Zhang et. al. structured an RNA editing system that can induce the A to inosine (I) replacement, this system can be used to edit full-length transcripts containing pathogenic mutations. It was shown that the RNA editing system is capable of robustly knockout genes and performing RNA editing in mammalian cells [95]. In 2019, they further upgraded this RNA editing system. The new system is named RESCUE, this new generation of RNA editing system kept the capacity of converting A to I, and it could also perform C-to-U and A-to-I editing [96]. In 2021, CRISPR C-to-G base editors for inducing targeted DNA transversions in human cells have been reported. The engineered base editors can efficiently induce targeted C-to-G base transversions in human cells [97]. All the new techniques mentioned above can be considered as promising tools for future personalized therapies against cancer because they can precisely modify single nucleotides, which maximize the possibilities for therapeutic genome editing. Recently, Lei et. al. reported that knocking out ATP‐binding cassette (ABC) B1 in colorectal cancer cells results in the restoration of the sensitivity to paclitaxel, suggesting that this approach is an promising way to deal with multidrug resistance [98].

On the top of that, CRISPR-Cas system has also been repurposed to perform epigenetic engineering [99]. For instance, accumulating studies have shown that manipulating the DNA-regulatory elements using CRISPR-Cas system is an efficient method to trigger epigenetic changes [100]. In particular, the CRISPR-dCas9 system contains a CRISPR guide and a variant of Cas9 protein whose endonuclease activity is lost. The CRIPSR-dCas9 serves as an ideal gene targeting platfrom for epigenetic modulators [101]. Moreover, cancer is associated with epigenetic changes such as aberrant histone modifications and DNA methylations [102]. In a previous study, dCas9 was linked with a epigenetic modulator which induces demethylation. This system induced demethylation A549 lung cancer cells with considerable efficiency [103]. In another study, the sgRNA-dCas9 targetng the protumor *Granulin* gene was conjugated with three different epigenetic suppressors: DNMT3a, KRAB, and EZH2. It was observed that, after treating the liver cancer cell Hep3B with the dCas9-epigenetic suppressor systems, the methylation level of the promoter for the target gene was elevated. Furthermore, the proliferation, invasion, tumor sphere formation the Hep3B were substantially inhibited by the suppressors [104].

In conclusion, genome editing is a straight-forward way to treat the diseases associated with genetic aberrations such as cancer. Mega-nuclease, ZFN, TALEN, and CRISPR-Cas9 are four major types of GETs. Although each of them has its pros and cons, efforts need to be devoted to improving the editing efficiency and minimalizing the off-targeting effect. Besides, except reconstructing and upgrading GETs themselves, designing and choosing appropriate delivery system is also a way to optimize the genome editing [105–107].

### Non-viral delivery platforms for genome editing in the treatment of cancer

GETs are made of proteins and nucleic acid, which makes it challenging to efficiently and safely deliver them into the cell nucleus [108]. Although viral vectors were widely used in laboratories and clinical trials to deliver genome editing therapies, the potential immunogenic issues surrounding viral vectors significantly hindered the translation of such therapies [109]. Nevertheless, non-viral delivery systems present themselves as a prospective choice. Although delivery efficiency is slightly lower than that of viral delivery systems [110], non-viral delivery systems could be artificially synthesized and are associated with fewer safety issues [111]. More importantly, the quick development in materials science and molecular biology may substantially facilitate the improvement of deliver efficiency of non-viral systems. In addition, the accumulation of the understanding about how cells uptake and process the non-viral vectors would also help researchers to develop more efficient delivery systems. So far, a variety of molecular modulators, including microtubules, Niemann-Pick type C protein 1, and Heat shock protein 70, have been reported to contribute to the delivery of non-viral vectors [112].

Generally, there are five major groups of non-viral delivery systems: peptide-based delivery system, lipid-based delivery system, inorganic delivery system, and polymeric delivery system, and electroporation. They have been used to deliver GETs for the direct editing of pathogenic cells in cancer therapies or ex vivo editing of immune cells. This section is focused on the current developments of these non-viral delivery systems in GET-based anti-cancer therapies. In addition, the advantages and limitations of this delivery system are also discussed.

#### Cell-penetrating peptide for the delivery of GETs into cancer cells

It has been well documented that cell-penetrating peptides (CPPs) can facilitate the cellular intake and uptake of molecules. Conjugating CPPs with therapeutic reagents could result in more efficient delivery [113]. Thus, they have been used as a delivery system for gene editing tools. It was demonstrated that CPPs could safely and effectively deliver ZFNs into cells. In addition, the cell-penetrating capacity of ZFN-CPP conjugates was observed to be much higher than that of ZFN alone [114]. It was also reported that the cell-penetrating poly-Arg peptide conjugating to a surface-exposed Cys residue present on each TAL effector repeat have superior cell-penetrating activity than that of purified TALEN proteins. This TALEN-CPP system was used to successfully knock out CCR5 and BMPR1A genes [115]. Further, Jain et. al. complexed CRISPR-Cas9 with an engineered, modular tandem peptide nanocomplex system. They showed that this CPP delivery system significantly contributes to the cell-targeting capacity and genome editing efficiency in OVCAR8 cells, HeLa cells, and 3TZ cells [116].

**Fig. 2:**
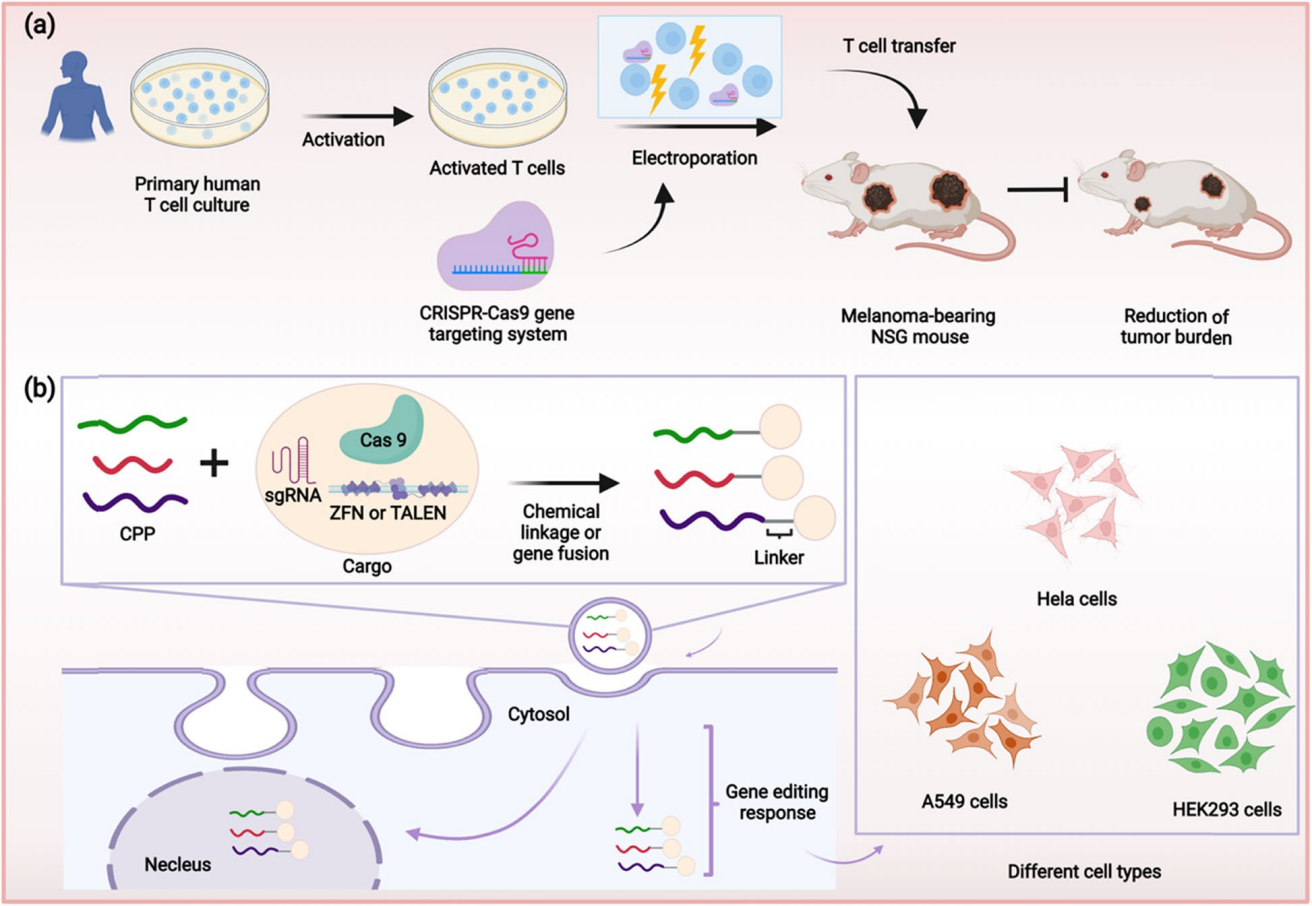
Schematic illustration of electroporation- and CPP-mediated delivery of GETs. **a** The activated human T cells reprogrammed by electroporation-mediated genome editing reduce the tumor burden of melanoma-bearing mice. **b** CPPs facilitate the genome editing in cancer cells which could be used to establish xenograft models

Inspired by the advantages of CPPs, they are also considered as a potential delivery system for personalized anti-cancer therapies. For example, an amphiphilic penetrating peptide has been synthesized by inducing the formation of hydrazone bond between a cationic peptide scaffold and a hydrophobic aldehyde tail. It was used to deliver Cas9 protein into human lung cancer A549 cells to delete the gene encoding hypoxanthine phosphoribosyltransferase 1 (Fig. 2b). The resultant high knockout efficiency indicates that CPPs can facilitate genome modification in cancer cells [117]. In another study, the CPP PTD4 was fused with an endosomolytic peptide CM18 and a 6x histidine-rich domain. The upgraded CPP-based non-viral delivery system was found to facilitate genome editing in different hard-to-modify cells including cancer cells including THP-1, Jurkat, and CA46 cells [118]. Additionally, CPP has been constructed into a self-assembling Cas9 ribonucleoprotein complex to act as a delivery system for genome-editing therapy against lung cancer. The self-assembling complex, named Cas9-LMWP comprises a nuclear localization sequence and a low-molecular-weight protamine (LMWP). This complex induced a 43.9 indels rates in KRAS gene of A549 lung cancer cells. Moreover, in xenograft lung cancer models established in female BALB/c nude mice, Cas9-LMWP exerted strong anti-cancer effects [119].

However, although CPPs present themselves as potential delivery systems for GETs, the intricate mechanism through which CPP enter cells is still unclear [120] and the clinical application of CPP is still hindered by the limited stability, selectivity and delivery efficiency [121]. Therefore, actions could be taken to explore solutions for such drawbacks.

#### Lipid-based delivery system for the delivery of GETs into cancer cells

Lipid nanoparticles are a clinically approved drug delivery system [122]. These spherical vesicles enclosed by a lipid bilayer membrane are biodegradable and biocompatible [123, 124]. However, the application of lipid-based systems for the delivery of GETs is hampered by shortcomings including limited half-life, stability, and encapsulation efficiency [125, 126]. The cationic lipids have been utilized to deliver Cas9-sgRNA nuclease complexes into cultured human cells and resulted in an 80% genome modification. Further, the cationic lipids also efficiently deliver different genome-editing proteins into the human cells and the mouse inner ear. The delivery of Cas9:sgRNA complexes led to a 80% genome modification *in vitro* and 20% *in vivo* [127]. As a carrier for therapeutic GET, cationic lipids were complexed with Cas9-sgRNA to treat autosomal dominant hearing loss in mice. The Cas9-sgRNA complex was engineered to target and disrupt the mutant Tmc1Bth allele. This strategy significantly reduced the progressive hearing loss, and substantially enhanced acoustic startle responses were observed in mice in treatment group. These findings suggest that the combination of lipid-based delivery system and GET may exert important roles in the treatment of the diseases caused by pathological gene mutations.

**Fig. 3:**
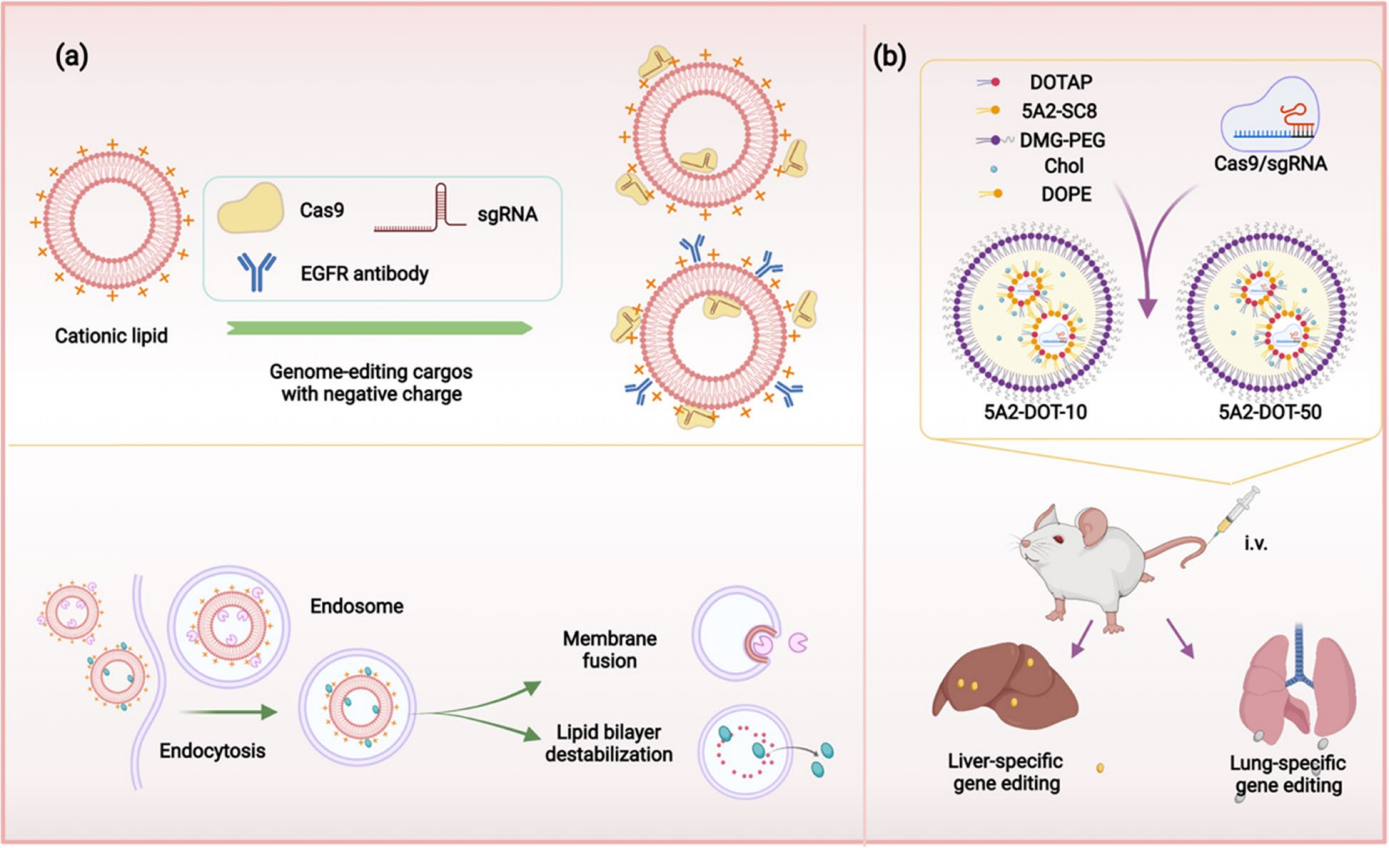
Schematic examples showing different applications of lipid-based delivery system for GETs. **a** CRISPR-Cas9 genome editing complexed with targeted lipid nanoparticles inhibit the growth of glioblastoma ovarian tumors. **b** Combing CRISPR-Cas9 with lipid nanoparticles for the tissue-specific gene editing or the generation of tumor models

In personalized anti-cancer therapies, several strategies have been applied to improve the delivery efficiency of lipid-based carriers [128]. It has been reported that, equipped with amino-ionizable lipid nanoparticles, Cas9 mRNA and sgRNAs targeting *PLK1* could be effectively delivered into aggressive orthotopic glioblastoma, and inhibit the tumor growth (Fig. 3a). By analyzing the tumors, it was found that up to 70% of gene editing had been achieved in Eight-week-old female C57BL/6JOlaHsd mice [122]. To be noticed, except developing personalized therapies according to the mutations identified in tumors and the genetic background of patients, establishing accurate cancer models that could recapitulate the characteristics the tumor in different patients is also an important step for designing personalized regimens and therapies. Daniel J. Siegwart and his colleague used their modified lipid nanoparticles as carriers for delivering Cas9-sgRNA ribonucleoprotein complexes into mouse tissues (Fig. 3b). Through this way, the Cas9-sgRNA ribonucleoprotein complexes could induce the cancer related mutations through editing the genome of cells. Consequently, organ-specific cancer models in livers and lungs were successfully generated [129]. Potentially, this approach can also be used to establish cancer models that recapitulate the patient-specific characteristics in mice, which is very helpful for the quick screening of drugs.

#### Inorganic vectors for the delivery of GETs into cancer cells

**Fig. 4:**
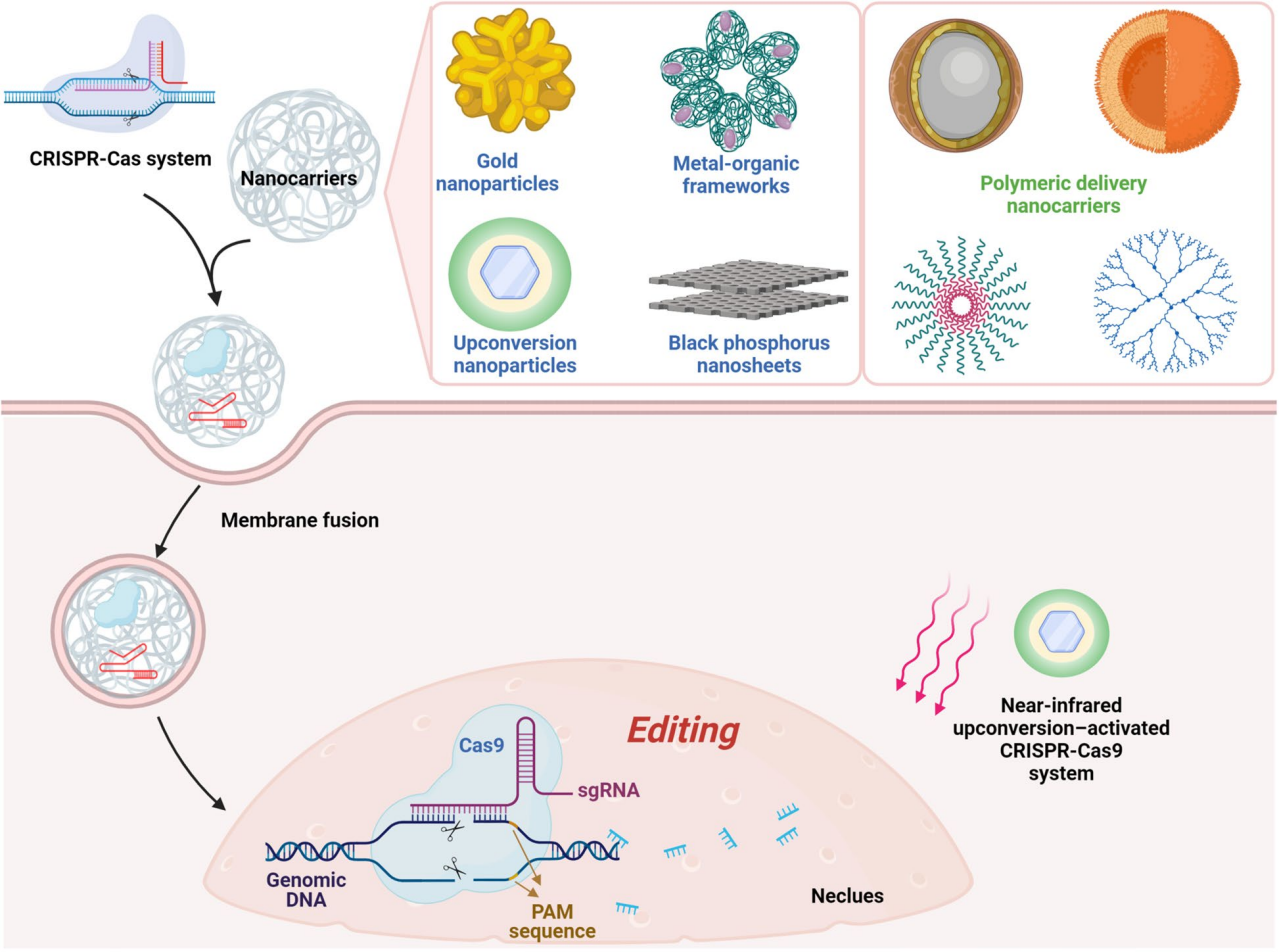
Schematic illustration of the inorganic and polymeric delivery systems for GETs

Armed with advantages including stability, biocompatibility and large loading capacity, inorganic vectors emerge as a novel drug delivery platform [130]. The recently popular inorganic delivery systems can be classified into four types: black phosphorus, graphene oxide, mesoporous silica nanoparticles, and gold nanoparticles [131–134]. More importantly, they are also employed to deliver GETs into cells (Fig. 4). For instance, gold nanoparticles possess a special ability to penetrate cell membranes. In addition, gold is generally well tolerated by the human body and can be easily conjugated with DNA. It was reported that Cas9-sgRNA was co-assembled with the cationic arginine gold nanoparticles gold nano-carriers and then delivered into Hela cells, human embryonic kidney cells (HEK-293T), and mouse macrophage (Raw 264.7) cells. As a result, around 90% Cas9-sgRNA complexes were delivered into the cells and led to successful genome editing with up to 30% efficiency [135]. In addition, the metal-organic frameworks represent another important delivery system based on inorganic vectors because they have sufficient surface area with adjustable shapes. Moreover, their porosities and biodegradability also make them ideal delivery systems for GETs. Khashab et. al. have developed a nanoscale zeolitic imidazole framework to deliver CRISPR-Cas9 into Chinese hamster ovary cells. It was demonstrated that the loading efficiency was 17% and after 4 days, the the yielded gene editing efficiency of this system was 37% [136]. Furthermore, black phosphorus nanosheets (BPs) is a new version of two-dimensional biomaterial for drug delivery. They have good element biocompatibility and are non-toxic [137]. Also, the proteins could tightly bind to the periodic atomic grooves on surfaces of BPs [138]. These properties make BPs suitable for delivering GETs. In a previous study, the engineered Cas9 ribonucleoproteins were loaded on BPs, and it was shown that BP system could deliver the Cas9 ribonucleoproteins into MCF-7 cells and resulted in 26.7-32.1% indel frenquency in vitro. In addtion, the administration of BP + engineered Cas9 ribonucleoproteins also led to the significant reduction of EGFP signals in A549/EGFP tumor-bearing nude mice [139].

Thus, the inorganic vectors are also implemented into genome editing therapies for personalized anti-cancer therapies. The metal-organic framework has been modified to facilitate the gene editing in cancer cells. To improve the cell-entering capacity of zeolitic imidazolate frameworks, a research group coated them with the membrane of cancer cells. This approach significantly increased the uptake of the zeolitic imidazolate frameworks encapsulating CRISPR-Cas9 by MCF-7 cells. In animal studies, it was further confirmed that the zeolitic imidazolate frameworks coated by the membrane of MCF7 tumor cells selectively accumulate in MCF7 cells [140]. Besides, up-conversion nanoparticles represent a novel inorganic delivery system which could convert near-infrared (NIR) light into local ultraviolet light, thus making it possible for the cleavage of photosensitive molecules in a controlled manner [141]. This strategy has been used to achieve the on-demand release of genome editing therapy against cancer. Song et. al. reported that they had complexed the up-conversion nanoparticles with CRISPR-Cas9 system targeting tumor gene polo-like kinase-1, and successfully turned on the gene editing using NIR light in 293T cells. As for the in vivo investigation, A549 cells were edited using the up-conversion nanoparticles and CRISPR-Cas9 system. Then, the cells were subcutaneously transplanted into the back of BALB/c mice, which resulted in 4.7 to 20.1% indel frequency after different duration of irradiation time [142]. This approach could potentially provide substantial feasibility and convenience to the genome editing in personalized anti-cancer therapies.

#### Polymeric delivery system for the delivery of GETs into cancer cells

Polymeric drug delivery system is defined as a polymeric formulation that transports the therapeutic substance into the cell or body. Accumulating evidence suggests that polymeric delivery systems are safe, efficient, and could stably control the rate, time, and place of drug release [143–147]. So far, a few types of polymeric systems have been used to deliver GETs (Fig. 4). They are boronic dendrimer [148], nano-clew [149], Cas9 micelles [150], Polyethylenimine complex [151], and polymeric nanocapsules [152]. All of them have been shown to improve the safety or efficiency of GETs. Since polymers have been widely used for drug delivery, their values are well characterized. Therefore, it is conceivable that these polymeric carriers would be quickly translated to clinically appliable delivery systems for therapeutics [153].

With respect to personalized anti-cancer therapy, several types of polymeric delivery systems were employed to improve the editing efficiency and safety of GETs due to their large packaging capacity and safety profile [154]. For example, Ping et. al. have constructed a supramolecular polymer system which allows the controlled delivery of Cas9 ribonucleoprotein targeting mutant KRAS in 293T cells and colorectal cancer cells [155]. By effectively disrupting mutant genes, tumor growth as well as metastasis in the BALB/c nude mice bearing SW-480 CRC cells were significantly inhibited. In addition, a chain-shattering polymeric nanoplatform has been recently developed to deliver the CRISPR-Cas9-based anti-cancer therapy. The chain-shattering polymeric nanoplatform is a trigger-responsive delivery system in which protecting groups regulate the unpack/release of the CRISPR-Cas9 targeting tumor gene EZH2. Armed with this delivery system, the genome editing efficiency of CRISPR-Cas9 in PC3 prostate cancer cell line reached 32.2%. In xenograft tumor model established in BALB/c nude mice (4–5 weeks old, 18–20 g), it resulted in a 21.3% editing efficiency and significantly inhibited tumor growth [156]. This new delivery method is very meaningful for personalized anti-cancer therapy because it provides considerable precision for the modulation of cancer related genes.

#### Electroportion for ex vivo editing of immune cells

Apart from complexing or conjugating GETs with some non-viral vectors, electroporation can directly increase the permeability of the cell membrane by hitting the cell membrane with electric pulses of sufficient intensity, allowing the penetration of GETs [157]. When the electroporation technique was first reported to be used for delivering GET, an editing frequency of 79% was observed in previously hard-to-transfect cells [158]. Later on, the procedure of electroporation was further optimized to reduce cell death [159]. This delivery system is convenient, effective and is suitable for mass production of genetically edited cells. Hence, it is currently the most used delivery system for ex vivo editing of immune cells [160, 161].

The electroporation technique provides a good platform for the mass production of chimeric antigen receptor (CAR) cells (Fig. 2a). CAR-T cell therapy is important personalized immunotherapeutic approach to treat cancer [162]. With this approach, the T cells could be reprogrammed by GET to effectively kill different types of cancers. Moreover, using genetically edited patient-derived T cells could largely avoid the host-verses-graft-diseases. Marson et. al. have reprogrammed the gene encoding the endogenous T cell receptor (TCR) to improve the cancer-targeting capacity of T cells. It was shown that the genetically edited T cells effectively recognize tumor antigens, leading to the suppression of tumor cell proliferation *in vitro* and inhibition of tumor growth *in vivo* [159]. Besides, electroporation-mediated genome editing can be also utilized in patient-derived stem cells which are considered as the cells of cancer origin. By inducing the key carcinogenic mutation in the cancer cell of origin, the personalized cancer models can be quickly established [163, 164].

Although convenient and effective, directly apply electroporation technique i*n vivo* is invasive, which means it is currently not an convinient option for delivering GETs into tumors as personalized gene therapies. However, substantial efforts have been deveoted to developing in vivo electroporation approaches for effective and safe delivery of therapeutic reagents. Recchia et. al. have successfully conducted the CRISPR-mediated the knockout of the RHO gene carrying the P23H mutation in the mouse retina through electroporation. To inject the DNA solution, the sclera was pierced with a 3-gauge needle. Additionally, the pores on cells were opened by five 90 V square pulses of 50 milliseconds duration [165]. For the treatment of tumor, in vivo electroporation has been used to deliver the gene emcoding anticancer protein such as IL-15 [166]. However, the possibility of using intratumoral electroporation for the delivery of GETs to correct neoplastic genes still repuires further explorations.

### GETs-based personalized anti-cancer therapies in clinical studies

Gene editing is a promising therapeutic option for the treatment of cancer, considerable efforts have been made to further elucidate the detailed mechanisms by which GETs target and modify human genes [167]. Moreover, the emerging of new generations of mega-nucleases, ZFNs, TALENs, and CRISPR-Cas9 systems have substantially enriched the toolbox for genome editing [168]. As a consequence, although genome editing is still relatively young in comparison to other cancer treatments, a lot of genome editing-based anti-cancer therapeutic methods have already gotten to the stage of clinical assessments. So far, 34 trials assessing the efficacy and safety of the genome editing-based anti-cancer therapies have been registered in the ClinicalTrials.gov database. Among them, the majority of studies are using genetically edited immune cells to fight against cancer. Also, given the fact that mega-nucleases, ZFNs, TALENs, and CRISPR-Cas9 systems have been all reported to efficiently perform genome editing in certain types of cells [169]. CRISPR-Cas9 systems are dominantly used in clinical studies. Notably, the clinical data from a phase I trial evaluating the safety and efficacy of reprogrammed T cells have been published. According to the report, CRISPR-Cas9 was used to knock out PD-1 gene in autologous T lymphocytes. These genetically edited T cells were then transplanted to patients with non-small cell lung cancer. When the trial was complete, no level 3-5 adverse events were observed. Moreover, two out of seven participants experienced stable disease with 17.6 and 22.0 weeks, the other five patients had progression disease. Thus, this cell therapy seems to be safe, but further studies with more rigorous evaluations are needed [170].

Additionally, the delivery systems for GETs of many trials are not specified in their ClinicalTrials.gov webpage. Among all the specified trials, 8 of them used non-viral delivery systems (Table 1). However, only one trial has the published data. In this trial, Cas9 and single guide RNA plasmids were transfected into patient derived T cells through electroporation to disrupt PD-1 gene. The edited T cells were used as immunotherapy for patients with refractory non-small-cell lung cancer. Among all the 12 patients who received the treatment, only grade 1/2 treatment-related adverse events occurred. Besides, the median progression-free survival and median overall survival were 7.7 weeks and 42.6 weeks [171]. These results suggest the encouraging safety and potential efficacy of the therapy. Furthermore, it is quite obvious that the current trend of non-viral delivery of CRISPR-Cas9 system is using electroporation. However, electroporation of T cells is associated with several limitations because it was reported that T cell viability, proliferation and gene expression could be affected by the electroporation procedure [172]. Hence, other non-viral delivery systems also hold promise for further facilitating the clinically applicable personalized anti-cancer therapies.

**Table 1:** Registered clinical trials using non-viral systems to deliver genome editing-based anticancer therapies

Delivery system	GET	Edited cell	Target Gene	Phase	NCT Number
Lipid Nanoparticles	CRISPR-Cas9	T cells	TCR	I/II	NCT05066165
Electroporation	CRISPR-Cas9	T cells	TCR, HLA-class I and HLA-class II	I	NCT05037669
Electroporation	CRISPR-Cas9	T cells	CD5	I	NCT04767308
Electroporation	CRISPR-Cas9	T cells	HPK1	I	NCT04037566
Electroporation	CRISPR-Cas9	T cells	TCR and PD-1	I	NCT03399448
Electroporation	CRISPR-Cas9	T cells	TCR and B2M	I/II	NCT03166878
Electroporation	CRISPR-Cas9	T cells	PD-1	I	NCT02793856
Plasmid in gel	CRISPR-Cas9 TALEN	Cancer cells	HPV E6/E7	I	NCT03057912

## Conclusions

Personalized therapies provide great hopes for patients with cancer. Genome editing is an ideal way to implement personalized therapies because it makes it possible to modulate pro-tumor genes or reprogram the anti-tumor immune cells. As the most advanced GETs, CRISPR-Cas9-based genome editing is very popular in the field of personalized therapy. Moreover, a variety of non-viral delivery systems including electroporation, CPP, lipid delivery system, inorganic vector, and polymeric delivery system have been used to transport GETs into the cell nucleus. Although it is generously believed that the delivery efficiency of non-viral delivery systems is relatively lower compared to viral delivery systems, they are safer and have better feasibility. Currently, several clinical studies are designed to assess the therapeutic values of genetically edited immune cells. Moreover, electroporation is dominantly used in these trials for the delivery of GETs. It is expectable that more translational studies will be conducted to further evaluate the potential of genome editing in personalized anti-cancer therapies. In addition, future studies may focus on the development of non-toxic, highly efficient non-viral vectors that could mediate the *in vivo* delivery of GETs.

## Data Availability

Not applicable.

## Abbreviations

GET: Genome editing tool
DSB: Double strand break
HDR: Homology-directed repair
NHEJ: Nonhomologous end joining
ZFN: Zinc-finger nuclease
TALEN: Transcription activator-like effector nuclease
CRISPR: Clustered regularly interspaced short palindromic repeats
HPV: Human papillomavirus
PD-1: Programmed cell death-1
TIL: Tumor infiltrating lymphocyte
TALE: Transcription activator-like effector
A: Adenine
C: Cytosine
G: Guanine
T: Thymine
TRL: Tumor-reactive lymphocyte
sgRNA: Single guide RNA
PAM: Protospacer adjacent motif
I: Inosine
CAR: Chimeric antigen receptor
TCR: T cell receptor
CPP: Cell-penetrating peptide
LMWP: Low-molecular-weight protamine
BP: Black phosphorus
NIR: Near infrared
